# Catalytic Combustion of Biodiesel Wastewater on Red Mud Catalyst

**DOI:** 10.3390/ma18030652

**Published:** 2025-02-01

**Authors:** Shangzhi Yu, Wenyu Yuan, Qinglong Xie, Xiaojiang Liang, Yong Nie

**Affiliations:** Biodiesel Laboratory of China Petroleum and Chemical Industry Federation, Zhejiang Province Key Laboratory of Biofuel, College of Chemical Engineering, Zhejiang University of Technology, Hangzhou 310014, China; ysz@zjut.edu.cn (S.Y.); yuanwyu@163.com (W.Y.); xieql@zjut.edu.cn (Q.X.); lxj0824@zjut.edu.cn (X.L.)

**Keywords:** red mud, catalytic combustion, biodiesel wastewater, Fe_2_O_3_, calcination temperature

## Abstract

The resource utilization of red mud (RM) has attracted widespread attention for achieving the waste-to-waste treatment goal. In this work, the RM catalysts were synthesized at different calcination temperatures by a simple method. The calcination temperature had a great effect on catalyst activity in the catalytic combustion of biodiesel wastewater. The RM catalyst calcined at 350 °C (RM350) exhibited the best catalytic activity. The chemical oxygen demand (COD) and COD removal rate of the treated wastewater reached almost 0 mg/L and 100%, respectively. The COD removal rate was significantly higher than 90.703% of the catalyst prepared by α-Fe_2_O_3_ at the same calcination temperature. Characterization results showed that the RM catalyst exhibited a high specific surface area of 60.03–64.15 m^2^/g and a well-developed mesoporous structure, as the calcination temperature did not exceed 400 °C, which was beneficial for adsorption and diffusion. Meanwhile, most of the Fe_2_O_3_ in the catalyst existed in an amorphous form and was abundantly presented on the catalyst surface, significantly lowering the reduction temperature of the catalyst and enhancing its reducibility. Furthermore, the α-Fe_2_O_3_ in the catalyst had higher dispersion, leading to an increase in utilization efficiency.

## 1. Introduction

Biodiesel, as a renewable energy, has attracted worldwide attention in recent years. With the increasing depletion of fossil fuel resources and the increasingly serious incidence of environmental pollution, biodiesel has become an important alternative to traditional fossil fuels due to its renewability and environmental protection [1,2,3]. However, a large amount of biodiesel wastewater will be produced during the production of biodiesel [4]. It is reported that approximately 0.2–3 L of biodiesel wastewater is produced for each liter of biodiesel production [5]. The amount of global biodiesel production in 2022 was close to 52 million tons [6], which means that more than 10.4 million tons of biodiesel wastewater were produced. Biodiesel wastewater has the characteristics of a complex composition, dark color, and strong odor. It contains a large number of organic compounds such as alcohols, organic acids, and esters, resulting in its chemical oxygen demand (COD) reaching tens of thousands to hundreds of thousands of mg/L [7,8,9,10]. Therefore, the efficient treatment of biodiesel wastewater faces great challenges. The treatment process of biodiesel wastewater mainly includes physical and chemical treatment, advanced oxidation treatment, electrochemical treatment, biological treatment, and multi-process combined treatment [5]. However, these processes have respective limitations, such as poor treatment performance, increased costs, and new environmental issues due to adding large quantities of chemical reagents, and complicated process flows. As the main organic compounds in biodiesel wastewater belong to volatile organic compounds (VOCs), they become VOC gases after heating and vaporization. Our group previously reported for the first time the use of catalytic combustion technology for the treatment of biodiesel wastewater. During the treatment process, the biodiesel wastewater was first heated and vaporized, and then successively passed through commercial Fe_2_O_3_ catalysts and Pt/Al_2_O_3_@cordierite catalysts for catalytic combustion [11,12]. This technology still showed excellent COD removal performance without the addition of chemical reagents or microorganisms. The COD of biodiesel wastewater with a COD of 99,465 mg/L can be reduced to 0 mg/L after treatment [13]. However, the use of noble metal catalysts and the two-step catalytic process in this technology lead to increased catalyst and energy costs. Therefore, it is necessary to further screen low-cost and high-catalytic activity catalysts and reduce the catalytic process.

Fe_2_O_3_, as a non-noble metal oxide, is more abundant, cheaper, and environmentally friendly than noble metals and other non-noble metal oxides. It exhibits excellent resistance to poisoning, good catalytic activity, and chemical stability in catalytic combustion applications [14,15,16,17]. Therefore, the catalyst with Fe_2_O_3_ as the active component in the field of catalytic combustion is very promising. Red mud (RM) as solid waste is a by-product of alumina production by the Bayer process, sintering process, or the combined process in the aluminum industry, which contains a large amount of Fe_2_O_3_ [18,19,20]. Until 2023, the global stock of RM exceeded 4 billion tons and continued to grow by 120 million tons annually [21]. In recent years, the design and development of catalysts using RM as solid waste had attracted the interest of researchers, as it not only realized the value-added of solid waste but also saved a lot of raw materials needed for catalyst preparation, which protected the environment and greatly reduced the cost of catalyst preparation. However, there were few reports on the preparation of catalytic combustion catalysts from RM. Moreover, though the calcination temperature was the key factor influencing the structure and properties of the catalyst, the researchers only studied the catalyst activity and other related properties at individual or several calcination temperatures not lower than 400 °C and did not systematically study the influence of a broader range of calcination temperatures on the structure, properties, and catalytic activity of the RM catalysts [22,23,24,25,26,27]. Therefore, as RM catalysts prepared at a high calcination temperature exhibit a low specific surface area and catalytic activity, researchers generally dissolve RM with hydrochloric acid, phosphoric acid, or oxalic acid, followed by precipitation with ammonia solution to improve the specific surface area and catalytic activity of the RM catalysts. Although these methods improve the catalytic activity of RM catalysts, they also significantly increase the cost, steps, and complexity of catalyst preparation. Meanwhile, the washing step with deionized water during the preparation process may also lead to new environmental problems. Therefore, it is necessary to systematically study the calcination temperature during the preparation of RM catalysts, along with an investigation of the activity and a detailed characterization of the catalysts to reveal the reasons that affect its catalytic activity and mechanical strength. To provide more references for the industrial application of RM catalysts, those mechanical strengths were investigated by preparing them into strips and testing the crushing strength.

This paper reported the preparation of the RM catalysts at different calcination temperatures by a simple method and their catalytic activity for the catalytic combustion of biodiesel wastewater. The effects of different calcination temperatures on the composition, pore structure, surface morphology, surface composition and oxidation states, and redox properties of the RM catalysts were characterized by a range of analytical techniques. The relationship between them and the catalytic activity and mechanical strength of RM catalysts was revealed, and compared with the catalyst prepared by using α-Fe_2_O_3_ instead of RM.

## 2. Experimental

### 2.1. Catalyst Preparation

A series of catalysts with high activity or high mechanical strength using RM as the active component were obtained by simple mixing, stirring, and extrusion operations. The detailed preparation process of the RM catalysts was described as follows: 100 g of RM (provided by Xinfa Group Co., Ltd., Liaocheng, China, 325 mesh) dried at 120 °C for 4 h and 2 g of kaolin (provided by China Kaolin Co., Ltd., Suzhou, China, 325 mesh) were mixed evenly first. Kaolin was used to increase the mechanical strength of the catalysts. Then, an appropriate amount of deionized water was added and mixed evenly. The strip catalysts with a diameter of 2 mm were obtained by the extrusion of the extruder, aged at room temperature for 2 h, and dried at 120 °C for 4 h. Finally, the samples were calcined at a heating rate of 5 °C/min at temperatures of 300 °C, 350 °C, 400 °C, 500 °C, 600 °C, 700 °C, 800 °C, 900 °C, 1000 °C, and 1100 °C for 6 h, respectively. The dimension of particle catalysts was controlled at a diameter of 2 mm and a length of 2–3 mm before experiments. According to the calcination temperature, a series of catalysts were expressed as RM300, RM350, RM400, RM500, RM600, RM700, RM800, RM900, RM1000, and RM1100, respectively. The RM catalyst without calcination was expressed as RM120. In addition, the Fe_2_O_3_ (AR, 325 mesh) purchased from Tianjin Zhiyuan Chemical Reagent Co., Ltd. (Tianjin, China) was used instead of RM to prepare a catalyst with a calcination temperature of 350 °C, which was expressed as Fe_2_O_3_-350.

### 2.2. Characterization

#### 2.2.1. Characterizations of Biodiesel Wastewater

The composition of biodiesel wastewater was determined by the area normalization method using gas chromatography–mass spectrometry (GC-MS). The relevant parameters and operating conditions of GC-MS were the same as those in the previous study, and the analysis accuracy was ±2% [28]. The gas after catalytic combustion was determined by gas chromatography (GC). The instrument parameters and operating conditions of GC are the same as those in the previous research [13]. The multi-parameter water quality analyzer (5b-6c (v8)) purchased from Beijing Lianhua Yongxing Technology Co., Ltd. (Beijing, China) was used to determine the COD of biodiesel wastewater by using the K_2_Cr_2_O_7_ method before and after the reaction, and the analysis accuracy was ±5%.

#### 2.2.2. Characterizations of Catalyst

The relative content of elements in the dried RM was determined by a wavelength dispersive X-ray fluorescence (XRF) spectrometer (ADVANT’X 4200) produced by Thermo Fisher Scientific (Waltham, MA, USA). X-rays were produced by the Rh anode. For quantitative analysis, a series of metal standards and UniQuant software (552, Thermo Fisher Scientific, Waltham, MA, USA) were used for calibration.

The samples were analyzed by a TGA/DSC 1 synchronous thermal analyzer (TGA/DSC) produced by Mettler Toledo, Stockholm, Sweden. The test temperature range was 25–1000 °C, and the heating rate was 10 °C/min.

The X-ray diffraction (XRD) patterns of the catalysts were obtained by an Empyrean Cu Kα radiation (λ = 0.1541 nm) X-ray diffractometer produced by Malvern Panalytical in Almelo, The Netherlands. The operating voltage and current were 40 kV and 40 mA, respectively. Patterns with a 2θ range from 5° to 80° were collected at a step of 0.01°.

The TENSOR II Fourier Transform Infrared Spectrometer (FTIR) produced by German Bruker Optics (Ettlingen, Germany) was used to record FTIR spectra in the air. The sample was mixed with KBr at a mass ratio of 1:100 and pressed into tablets.

The surface morphology of the catalysts was measured by a VEGA 3 scanning electron microscope (SEM) produced by TESCAN, Brno, Czech Republic, and the accelerating voltage was 30 kV.

The specific surface area and pore structure analyzer (3H-2000PS1) produced by Best Instrument Technology (Beijing) Co., Ltd. (Beijing, China) was used to determine the structural parameters of the catalysts. Nitrogen was used as the adsorbate, and the degassing temperature and time were 200 °C and 180 min, respectively. The analytical accuracy of the instrument was ±1%.

X-ray photoelectron spectroscopy (XPS) was recorded on a Thermo Scientific K-Alpha spectrometer using an Al Kα (1486.6 eV) radiation source operating at 12 kV and 6 mA. The narrow-spectrum scanning step was 0.1 eV, and the binding energy was calibrated according to the C 1s peak at 284.8 eV.

A H_2_ temperature-programmed reduction (H_2_-TPR) was performed using an AutoChem II 2920 device produced by Micromeritics, Norcross, GA, USA. About 150 mg of the sample was placed in a reaction tube, heated from room temperature to 120 °C at a heating rate of 10 °C/min under Ar gas flow (30 mL/min), dried for 60 min, and then cooled to 50 °C. After the baseline was stabilized by a 10% H_2_/90% Ar mixture (50 mL/min) for 0.5 h, the temperature was increased to 800 °C at a heating rate of 10 °C/min under a 10% H_2_/90% Ar (30 mL/min) atmosphere, and the signal was detected by a thermal conductivity detector (TCD).

According to the GB/T 30202.3-2013 standard from China [29], the KC-3AT automatic particle strength tester (Taizhou AOPUTE analytical instrument Co., Ltd., Taizhou, China) was used to radially squeeze the catalyst particles with a diameter of 2 mm and a length of 2.2 ± 0.1 mm to obtain the mechanical strength of the catalyst. The mechanical strength expressed by the radial crushing strength (P = F/L, P (N/cm) is the radial crushing strength, F is the pressure value (N), and L is the catalyst length (cm)).

### 2.3. Catalytic Test

The catalytic combustion reaction was carried out using a fixed bed flow reactor. The reactor consists of two vertical glass tubes with an inner diameter of 2.2 cm and a length of 22 cm. The upper section was the preheating section and the lower section was the catalytic combustion section. About 70 g of silicon carbide (SiC) and 20 g of catalyst were loaded into the preheating section and the catalytic combustion section, respectively. The temperature of the preheating section and the catalytic combustion section can be stabilized at 320 °C by the electric heating, which was controlled by the K-type thermocouple automatic switch with a control accuracy of ±1 °C. Biodiesel wastewater and air continuously entered the reactor from the top of the preheating section at a flow rate of 15 g/h and 800 mL/min, respectively, in which the air as the source of oxygen was excessive. The wastewater and air were heated in the preheating section, and the wastewater was fully mixed with the hot air after vaporization and entered the catalytic combustion section for reaction. The gas after the reaction was condensed into a liquid by a condensing tube and collected into a flask, and the uncondensed gas was passed into an absorption flask containing NaOH solution. The liquid and gas products were collected every 30 min for offline analysis, and the catalytic activity of the catalyst was reflected by the COD and COD removal rate of the wastewater after the reaction. The calculation formula of the COD removal rate was as follows:

$$COD\;removal\;rate=\left(1-\frac{COD\;of\;treated\;wastewater}{COD\;of\;untreated\;wastewater}\right)\times 100\%$$

## 3. Results and Discussion

### 3.1. Analysis of Biodiesel Wastewater

The composition of biodiesel wastewater was complex, and the main components were esters and alcohols. The physicochemical properties of biodiesel wastewater are shown in Table 1. As listed in Table 1, the COD of biodiesel wastewater diluted by deionized water was 114,500 ± 7600 mg/L, and the biodegradability of the wastewater was general according to the BOD_5_ value. In addition, the conductivity value showed that the total content of salts, electrolytes, and other conductive substances in biodiesel wastewater was low. The colority value and the color of the wastewater indicated that the biodiesel wastewater had a dark color.

### 3.2. Composition of RM

The RM used in this study was produced by the Bayer process for preparing alumina. The relative mass fraction of each element in the dried RM is shown in Table 2. The composition of the RM was similar to that in other reports. The largest element was Fe, reaching 27.74 wt%, which mainly existed in the form of the active component Fe_2_O_3_ [22,24,30]. In addition, Al and Si were usually present in the form of inert oxides Al_2_O_3_ and SiO_2_, respectively [31,32,33]. They could improve the water resistance, specific surface area, and dispersion of active components of the catalyst [34,35]. Na in RM commonly existed in the form of Na_2_O [36]. The presence of Na might have enhanced the sintering of the catalyst and inhibited the activation process of the active component Fe_2_O_3_ [22,37]. Ti usually existed in the form of TiO_2_ in RM. TiO_2_ was commonly used as a carrier in the catalyst, which showed a positive effect on the enhancement of catalyst activity [33,38]. Ca, S, K, and P were considered unwanted components in catalysts [22,24,39]. Although Mg existed in RM in the form of inert oxide MgO, the presence of MgO may have improved the dispersion of the active component Fe_2_O_3_ in RM, and the interaction with Fe_2_O_3_ increased the length of the Fe-O bond, which played an activating role in the release of O atoms from Fe_2_O_3_ [36,40]. The V element in RM usually existed in the form of active oxide V_2_O_5_. Studies have shown that V_2_O_5_ could not only improve the dispersion of the active components in the catalyst but also induce the generation of more surface chemical oxygen [41,42].

### 3.3. TG-DSC

**Figure 1:**
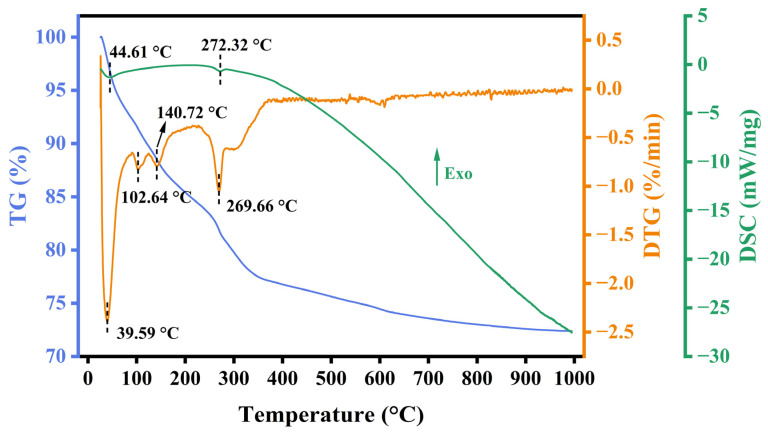
TG, DTG, and DSC curves of RM120.

To explore the thermal stability, composition change, and decomposition behavior of the RM catalyst at different temperatures, the uncalcined RM120 catalyst was analyzed by TG, DTG, and DSC, and the results are shown in Figure 1. The total mass loss of the catalyst was 27.62% while it had lost 23.23% before 400 °C, which indicated that the catalyst had good thermal stability as the temperature was higher than 400 °C. The DTG curve revealed four distinct weight loss peaks for RM120, located at 39.59 °C, 102.64 °C, 140.72 °C, and 269.66 °C. The weight loss peak at 39.59 °C was attributed to the desorption of gas adsorbed physically, which was proved by the endothermic peak of the DSC curve at 44.61 °C. The weight loss peaks at 102.64 °C and 140.72 °C were due to the volatilization of free water in the catalyst [43]. The weight loss peak at 269.66 °C was attributed to the decomposition of Al(OH)_3_ [44], which could be proved by the endothermic peak of DSC at 272.32 °C and the existence and disappearance of the Al(OH)_3_ phase in RM120 and RM300 in the next section of XRD analysis. At lower temperatures, the decomposition of Al(OH)_3_ will generate γ-Al_2_O_3_ with a porous structure, which contributes to the increase in the specific surface area of the RM catalysts.

### 3.4. Crystal Phase Composition

**Figure 2:**
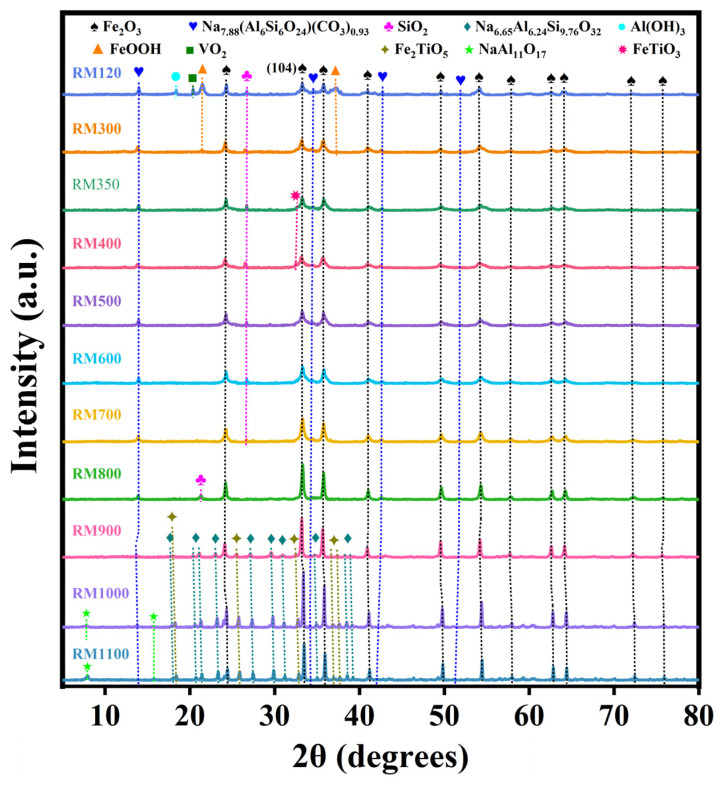
XRD pattern of the as-prepared RM catalysts.

The crystal phase composition of the RM catalysts was analyzed by XRD, as shown in Figure 2. The diffraction peaks of α-Fe_2_O_3_ (Hematite, JCPDS No. 79-1741) and Na_7.88_(Al_6_Si_6_O_24_) (CO_3_)_0.93_ (Sodalite, JCPDS No. 89-9098) were found in all catalysts. As the calcination temperature did not exceed 400 °C, the intensity of the diffraction peaks of α-Fe_2_O_3_ remained nearly unchanged, and the crystallite size was relatively small (Table 3). Within the calcination temperature range of 500 °C to 1000 °C, the peak intensity gradually increased, and the peak became higher and narrower from 700 °C. This indicated that as the calcination temperature was not higher than 400 °C, most of the Fe_2_O_3_ in the catalyst existed in an amorphous form and the dispersion of α-Fe_2_O_3_ was higher. Starting at 500 °C, the increase in the long-range order of α-Fe_2_O_3_ in the catalyst led to an increase in crystallinity. From 700 °C, the crystallite size of α-Fe_2_O_3_ increased significantly (Table 3). RM1000 calcined at 1000 °C had the largest diffraction peak intensity and grain size of α-Fe_2_O_3_ compared with other catalysts. In addition, SiO_2_ (Quartz, JCPDS No. 79-1960) existed in the catalyst as the calcination temperature did not exceed 700 °C and disappeared at 800 °C, but a new SiO_2_ (Cristobalite, JCPDS No. 85-0621) was formed. With the further increase in calcination temperature, Na_6.65_Al_6.24_Si_9.76_O_32_ (Nepheline, JCPDS No. 83-2372) and Fe_2_TiO_5_ (Pseudobrookite, JCPDS No. 73-1631) were found at 900 °C, and NaAl_11_O_17_ (Diaoyudaoite, JCPDS No. 21-1096) was found at 1000 °C. This indicated that SiO_2_ changed with the increase in calcination temperature and combined with other components to produce new crystal phases under the effect of a high temperature. In addition, the appearance of more stable new crystal phases at high temperatures also meant that the components appeared to fuse, indicating that the structure of the catalyst became more compact. This was the key factor leading to the improvement of the mechanical strength of the RM catalysts. FeOOH (Goethite, JCPDS No. 81-0464), Al(OH)_3_ (Gibbsite, JCPDS No. 76-1782) and VO_2_ (Paramontroseite, JCPDS No. 73-0514) were also present in the uncalcined RM120 catalyst. However, as the calcination temperature reached 300 °C, the peak intensity of FeOOH decreased rapidly, while the crystal phases of Al(OH)_3_ and VO_2_ disappeared. At 350 °C, FeOOH was completely decomposed, but FeTiO_3_ (Ilmenite, JCPDS No. 75-0519) was produced. As the calcination temperature reached 500 °C, the crystal phase disappeared.

### 3.5. FTIR Spectra

**Figure 3:**
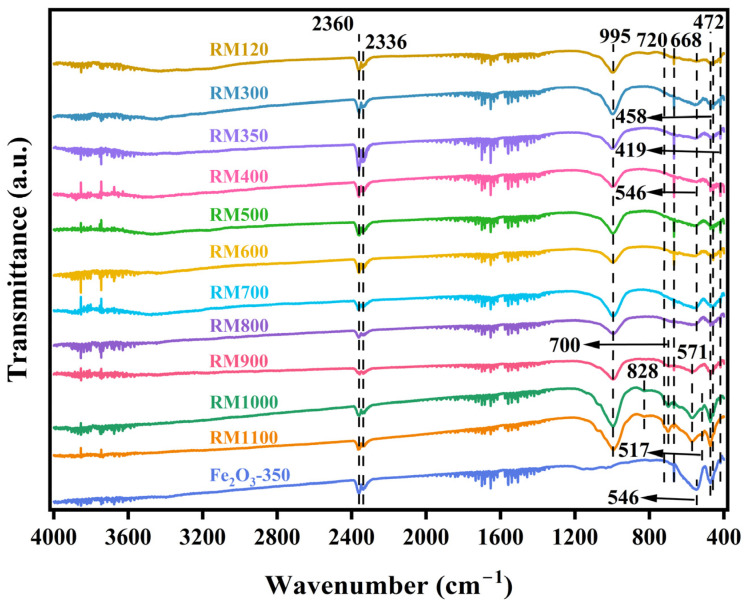
FTIR spectra of the as-prepared catalysts.

Figure 3 shows the FTIR spectra of the as-prepared catalysts. The peaks near 2360 cm^−1^ and 2336 cm^−1^ in all catalysts were caused by the antisymmetric stretching vibration of the C=O bond in CO_2_ [45]. Among them, the peak intensity of RM350 was the largest, which indicated that the catalyst had the strongest adsorption effect on CO_2_. This indicated that the gas desorption weight loss peak at 39.59 °C in TG-DSC analysis was likely to be caused by the desorption of CO_2_. The absorption band near 995 cm^−1^ was attributed to the stretching vibration of the Si-O bond in SiO_2_ [43,46]. Combined with XRF and XRD analysis, most of the SiO_2_ in the RM catalysts existed in the amorphous form. RM1000 and RM1100 contained more SiO_2_ than other catalysts, which indicated that the substance containing the Si element in the catalyst will decompose to form SiO_2_ under high temperatures. According to XRD analysis, the peaks near 828 cm^−1^ and 517 cm^−1^ in RM1000 and RM1100 might have been caused by NaAl_11_O_17_. The absorption bands near 720 cm^−1^ and 546 cm^−1^ were caused by Si-O-Al [47], while the absorption band near 668 cm^−1^ was caused by the bending vibration of Si-O [48]. The peak near 458 cm^−1^ reflected the Si-O-Si bending vibration in the SiO_4_ tetrahedral structure [49]. A dense band attributed to the bending vibration of Al-O was observed near 419 cm^−1^. The above five peaks were caused by kaolin added during the preparation of the catalyst. The peak near 700 cm^−1^ may have been caused by Fe_2_TiO_5_. The absorption band near 571 cm^−1^ was caused by the symmetric stretching of the Si-O-Al framework [50]. Combined with XRD analysis, this was caused by Na_6.65_Al_6.24_Si_9.76_O_32_. The peak near 472 cm^−1^ indicated the stretching vibration of the Fe-O bond [51]. For the red mud catalyst, the peak intensities in RM1000 and RM1100 were larger than those in the catalyst at other calcination temperatures but less than those in Fe_2_O_3_-350. According to the XRD patterns, the increase in the peak intensity was related to the increase in the crystallinity of α-Fe_2_O_3_.

### 3.6. Catalyst Morphology

**Figure 4:**
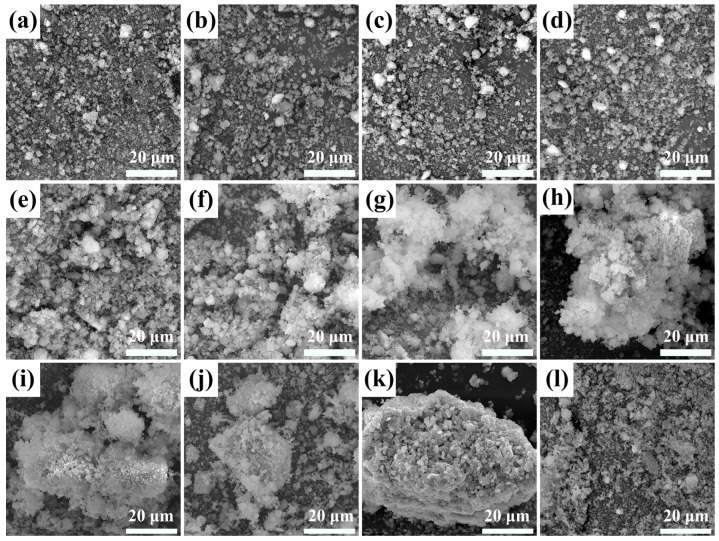
SEM images of fresh (**a**) RM120, (**b**) RM300, (**c**) RM350, (**d**) RM400, (**e**) RM500, (**f**) RM600, (**g**) RM700, (**h**) RM800, (**i**) RM900, (**j**) RM1000, (**k**) RM1100, and (**l**) Fe_2_O_3_-350.

The morphology of the as-prepared catalysts was studied by SEM. Typical SEM images are shown in Figure 4. In the RM120, most of the particle sizes were smaller than 2 μm, and there was a certain proportion of spherical particles, but most of them existed in the form of small fragments or other irregular particles (Figure 4a). As the calcination temperature did not exceed 400 °C, the morphology of the catalysts was similar to RM120, and no agglomeration or sintering was found, which indicated that the catalyst may have a higher specific surface area (Figure 4b–d). However, as the calcination temperature increased to 500 °C, the particles began to agglomerate and sinter. Smaller fragments started to adsorb on larger particles. Moreover, many spherical particles smaller than 1 μm attached to the large particles, which was caused by the growth of small fragments, and this also led to the increase in the crystallinity of the α-Fe_2_O_3_ crystal phase (Figure 2 and Figure 4e). As the calcination temperature continued to increase, the particles of RM600 tended to bond and form large particles, but the overall structure was loose. In addition, many small flocculent structures were formed on the surface of the particles (Figure 4f). In RM700, the sintering phenomenon was intensified, which led to a significant increase in crystallite size (Table 3). The overall structure of the catalyst was more compact, and the flocculent structure also increased (Figure 4g). For RM800, the aggregate was formed by further sintering of the particles, and the bonding between the small particles on its surface was also tighter. Notably, the connection between particles through flocculent structures could be observed on the surface of the aggregate (Figure 4h). This showed that the aggregate was formed by the continuous connection of the flocculent structures with particles and further interaction at high temperatures during the sintering process. As the calcination temperature reached 900 °C, the flocculent structure was reduced, which may be the reason that most of the small fragments had been sintered to form larger particles at this temperature (Figure 4i). In RM1000, the flocculent structure almost disappeared, and the particle size forming the aggregate was relatively uniform, indicating that particle size had changed under high temperatures. Combined with the XRD results, the Fe_2_O_3_ in the catalyst had been highly crystallized at this time (Figure 2 and Figure 4j). As the calcination temperature rose to 1100 °C, the sintering of the catalyst was very serious, the surface of the aggregate was very dense, and there was a mutual fusion between the particles, which may have been caused by the temperature being close to the melting point of the mixture (Figure 4k). In this case, the crystal phase was easy to change, which is the reason for the decrease in the peak intensity of the α-Fe_2_O_3_ (Figure 2). In Fe_2_O_3_-350, it was mainly composed of small fragments, and the overall structure formed was loose (Figure 4l). According to the above analysis, as the calcination temperature was lower than 500 °C, the mechanical strength of the catalyst was low. As the calcination temperature increased, the sintering of the catalyst led to an increase in mechanical strength, and the increase was greater within 900–1100 °C.

### 3.7. Pore Structure Analysis

**Figure 5:**
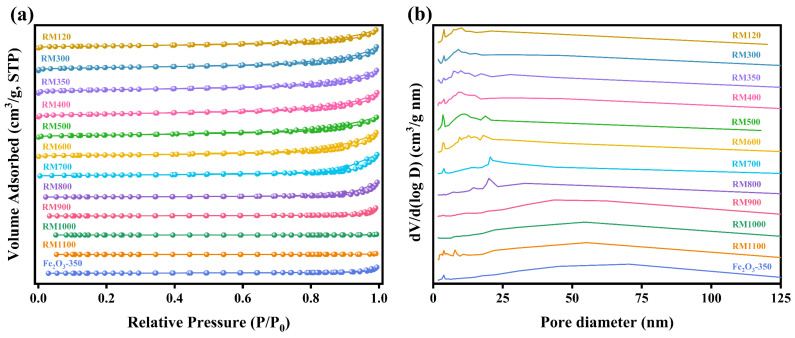
(**a**) N_2_ adsorption–desorption isotherms and (**b**) pore-size distribution curves of the as-prepared catalysts.

The microstructure characteristics of the as-prepared catalysts were investigated by N_2_ adsorption–desorption isotherms, and the results are shown in Figure 5 and Table 3. From Figure 5a, a typical type IV isotherm was obtained for the as-prepared catalysts, and the H3-type hysteresis loop was shown in the relative pressure range of 0.4–1.0 (P/P_0_), which was the characteristic of mesoporous materials. As the calcination temperature exceeded 700 °C, the N_2_ adsorption capacity of the catalyst decreased significantly, indicating a substantial decrease in pore volume and a significant increase in the average pore diameter (Table 3). By observing Figure 5b, as the calcination temperature did not exceed 400 °C, the pore size in the catalyst was mainly concentrated in the range of 1–17 nm, and this suitable pore size increased the specific surface area of the catalyst. As the calcination temperature increased to 500 °C, the proportion of pores with a pore size of 2–3 nm decreased, while the proportion of pores with a pore size of 3–4 nm increased. This further indicated that the crystallites began to grow at this calcination temperature. As the calcination temperature continued to increase, the pore size tended to increase gradually.

The BET surface area of the as-prepared catalysts was in the range of 0.65–64.15 m^2^/g and RM350 had the highest value. The higher specific surface area of the catalyst indicated more available active sites, suggesting that RM350 may exhibit the best catalytic activity. For the RM300, the specific surface area increased from 43.37 m^2^/g to 61.55 m^2^/g compared to the RM120, with an increase of 41.92%. According to the TG-DSC analysis, the increase in specific surface area was due to the decomposition of Al(OH)_3_ and the removal of water. As the calcination temperature rose to 400 °C, the specific surface area of the catalyst decreased slightly to 60.03 m^2^/g. With the further increase in calcination temperature, the specific surface area of the catalyst began to decrease rapidly. This was due to the catalyst sintering and the growth of the crystal grains, combined with XRD and SEM results. The maximum decrease in the specific surface area reached 23.20%, as the calcination temperature increased from 400 °C to 500 °C. As listed in Table 3, the significant decrease in specific surface area was mainly due to the decrease in pore volume caused by sintering. The increase in pore volume of RM600 could be attributed to pore merging under high temperatures. Overall, RM300, RM350, and RM400 exhibited relatively high specific surface areas (60.03–64.15 m^2^/g) while maintaining large pore volumes (0.153–0.164 m^3^/g). This indicated that the pore structure of the catalyst had been highly developed in the calcination temperature range of 300–400 °C, which was conducive to adsorption and diffusion.

### 3.8. Surface Chemical Composition and State

**Figure 6:**
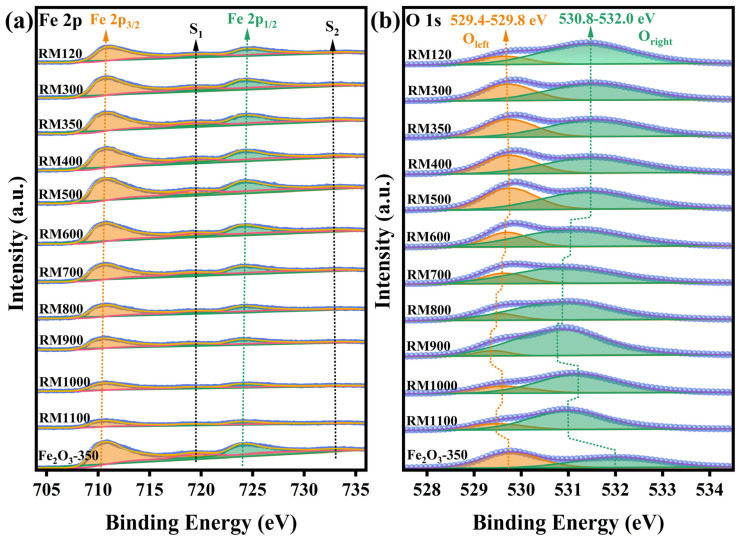
(**a**) Fe 2p and (**b**) O 1s XPS spectra of the as-prepared catalysts.

The surface composition and chemical state of the as-prepared catalysts were studied by XPS, and the spectra are shown in Figure 6. Figure 6a showed that the Fe 2p region consisted of two sub-peaks, Fe 2p_3/2_ and Fe 2p_1/2_, and their respective satellite peaks S_1_ and S_2_, which were generated by the spin–orbit coupling effect-splitting of Fe 2p orbital electrons. The peak fitting of the Fe 2p region using the nonlinear least squares (NLS) method found the presence of Fe^3+^, but no Fe^2+^ or Fe was detected. The peak information of Fe 2p_3/2_ and Fe 2p_1/2_ was obtained by Voigt function fitting and summarized in Table 3. By calculation, the splitting energy between Fe 2p_1/2_ and Fe 2p_3/2_ in the Fe 2p region of all catalysts was 13.5 eV, which was the characteristic of the existence of the Fe^3+^ oxidation state [52,53,54]. The satellite peaks S_1_ and S_2_ with binding energies around 719.3 eV and 733.3 eV were also attributed to the oxidation state of Fe^3+^. In general, the higher the oxidation state of Fe, the higher the binding energy. The peak binding energies of Fe 2p of the RM catalysts were not lower than Fe_2_O_3_-350, which further indicated that there was only an Fe^3+^ oxidation state on the surface of the RM catalysts. It also showed that the interaction between the components in the RM catalysts increased the peak binding energy, and the interaction was more significant as the calcination temperature was below 500 °C. Overall, the peak intensity of the Fe 2p region gradually increased in the calcination temperature range of 300–500 °C, indicating that the number of Fe^3+^ on the catalyst surface increased. It meant that more Fe^3+^ could participate in the redox cycle and promote electron transfer, thereby accelerating the reaction rate and improving the activity of the RM catalysts. As the calcination temperature continued to increase, the peak intensity of the Fe 2p region rapidly weakened. Combined with XRD analysis, as the calcination temperature was lower than 500 °C, the amorphous and microcrystalline Fe species in the catalyst were transformed into crystal phases and grew to form larger crystal grains with the increase in temperature, respectively. In the process of structural rearrangement and crystal growth, Fe atoms migrated from the bulk phase to the surface to form a more stable crystal plane. This led to more Fe atoms being exposed to the catalyst surface, thereby increasing the intensity of the Fe 2p region peak. As the calcination temperature continued to rise from 500 °C, the increase in the crystallite size, the decrease in the surface area, and the coverage of the catalyst surface by other substances all resulted in the decrease in the number of Fe atoms exposed on the catalyst surface, so that the intensity of the Fe 2p peak in XPS was rapidly weakened.

The O 1s region shown in Figure 6b was deconvoluted into two peaks. The left peak (O_left_) in the peak center binding energy range of 529.4–529.8 eV was attributed to metal oxides, namely metal-oxygen, while the right peak (O_right_) in the peak center binding energy range of 530.8–532.0 eV was attributed to surface adsorbed oxygen and silicates, aluminates, carbonates, and other inert substances with higher binding energy. For O_left_, its intensity gradually increased from RM120 to RM500 and then decreased rapidly from RM500 to RM900. The change trend was very similar to the Fe 2p peak. According to the binding energy of O_left_, the peak was mainly attributed to the metal-oxygen of Fe_2_O_3_ [15,16,52,55]. The increase in the amount of metal-oxygen improved the oxidative ability of the catalyst, thus exhibiting better catalytic performance. Notably, as the calcination temperature was higher than 500 °C, the peak in the O 1s region shifted significantly, indicating that the substances on the catalyst surface had changed, which suggested that the Fe^3+^ oxide was covered.

### 3.9. Reducibility

**Figure 7:**
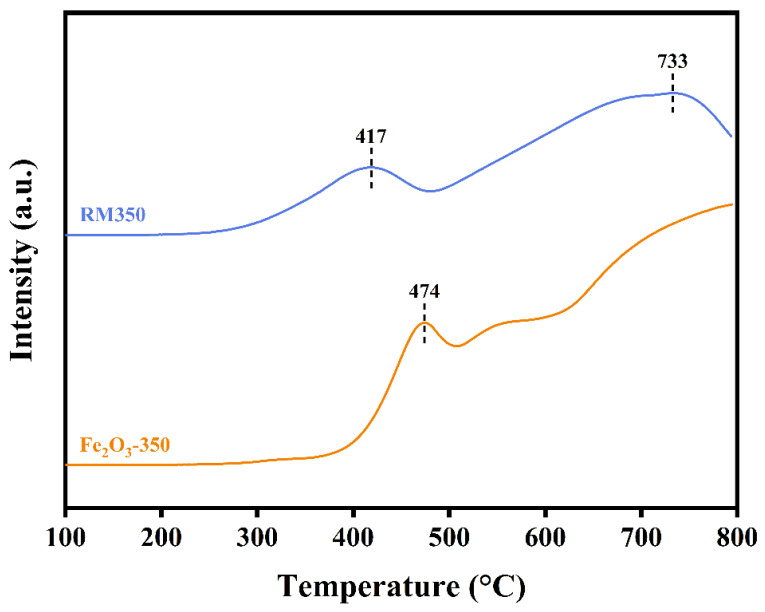
H_2_-TPR profiles of RM350 and Fe_2_O_3_-350.

The H_2_-TPR profiles of RM350 and Fe_2_O_3_-350 are shown in Figure 7. Two obvious reduction peaks were observed in RM350, and the peak temperatures were 417 °C and 733 °C, respectively. In contrast, only one peak at 474 °C was observed in Fe_2_O_3_-350, and the peak temperature of the next peak exceeded 800 °C. The existence of these peaks was closely related to the continuous reduction of Fe_2_O_3_ to Fe_3_O_4_, Fe_3_O_4_ to FeO, and FeO to Fe [56]. The peak temperature of the first reduction peak of RM350 was 57 °C lower than that of Fe_2_O_3_-350, and the reduction phenomenon of RM350 had begun to appear near 250 °C. This indicated that RM350 had better low-temperature reducibility, and had more reducible and mobile metal-oxygen than Fe_2_O_3_-350 at low temperatures. It also proved that the migration efficiency of metal-oxygen in RM350 was higher at low temperatures. Higher oxygen migration efficiency meant a higher amount of catalytic activity. Combined with XRD analysis and XPS results, the RM350 had a lower temperature reduction peak than Fe_2_O_3_-350 because of the more active amorphous Fe_2_O_3_ in RM350 and the interaction between components.

### 3.10. Catalytic Activity and Mechanical Strength

**Figure 8:**
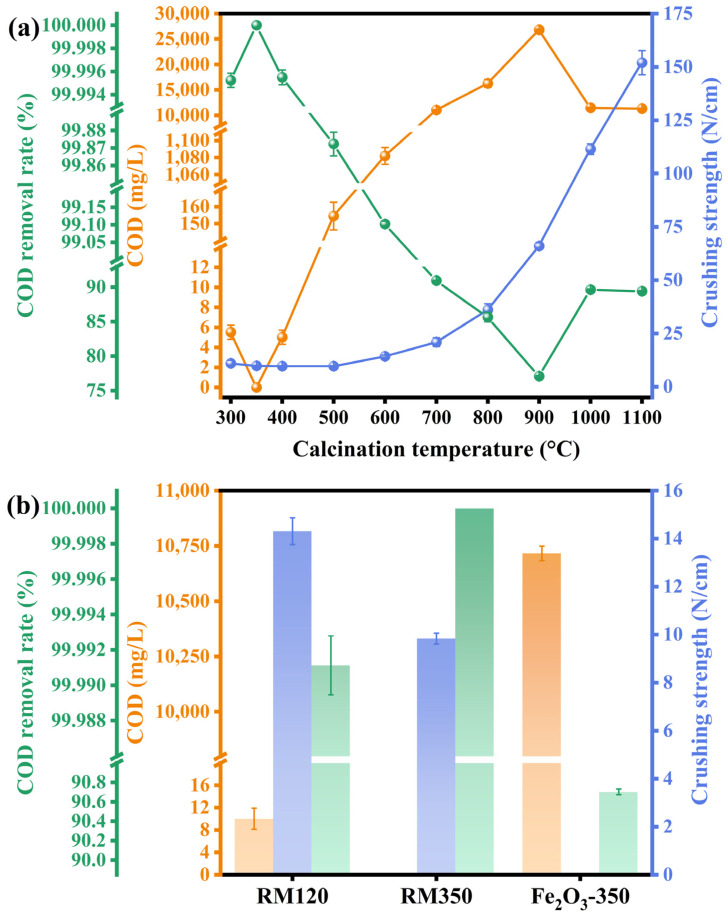
The mechanical strength of (**a**) the catalyst at different calcination temperatures and (**b**) RM120, RM350, and Fe_2_O_3_-350 and the COD and COD removal rate of biodiesel wastewater after catalytic combustion.

The catalytic activity and mechanical strength were reflected by the COD of the wastewater after catalytic combustion treatment, the COD removal rate, and the radial crushing strength of the catalysts. The results are shown in Figure 8. From Figure 8a, in terms of catalytic activity, RM350 showed the best COD removal effect, with the COD of the treated biodiesel wastewater being 0 mg/L and the COD removal rate being as high as 100%. In addition, GC analysis of the gas after the reaction did not find the presence of organic compounds, indicating that the organic compounds in the wastewater were converted into CO_2_ and H_2_O. As the calcination temperature did not exceed 400 °C, the catalyst had excellent catalytic activity, and the COD removal rate was higher than 99.996%. For RM500, the COD of the treated wastewater was 154.5 mg/L, and the COD removal rate was 99.872%. The treated wastewater could meet the wastewater discharge standard of China [57]. For RM600, the COD of treated wastewater reached 1082 mg/L, and the COD removal rate was 99.102%, which could not meet the standard. As the calcination temperature continued to increase, the catalytic activity of the catalyst decreased significantly. The COD of the treated wastewater exceeded 10,000 mg/L, and the COD removal rate was below 91%. In terms of mechanical strength, in the calcination temperature range of 300–500 °C, the crushing strength of the catalysts was less than 12 N/cm. With the increase in calcination temperature, the mechanical strength significantly increased from 700 °C. The crushing strengths of the RM800, RM900, RM1000, and RM1100 catalysts were 36.11, 66.06, 111.51, and 152 N/cm, respectively. The increase in crushing strength was 68.80%, as the calcination temperature increased from 900 °C to 1000 °C.

Figure 8b compared the mechanical strength of RM120, RM350, and Fe_2_O_3_-350 and the COD and COD removal rates of the treated wastewater. The highest catalytic activity of the three catalysts was that of RM350. For the uncalcined RM120, the COD of the treated wastewater was 10 mg/L, and the COD removal rate reached 99.991%, which also showed an excellent removal effect. However, the catalytic combustion performance of Fe_2_O_3_-350 was not ideal. The COD of the treated wastewater was 10,717 mg/L, the COD removal rate was 90.703%, and the strength of the catalyst was lower than the detection limit of the instrument.

According to the experimental results in this section and the results and analysis of the previous related characterizations, the specific surface area was a key factor affecting the activity of the catalysts. The larger specific surface area meant more active sites. Though the specific surface area of RM120 was lower than that of RM500, the catalytic activity of RM120 was better than that of RM500. This was because RM120 was calcinated at the reaction temperature of 320 °C, which led to the increase in the specific surface area. Although RM500 had more surface Fe^3+^ and metal-oxygen than RM300, RM350, and RM400, its catalytic activity was lower. This was because the sintering led to a decrease in the specific surface area of RM500, which reduced the active sites. As the calcination temperature increased, the specific surface area and the amount of Fe^3+^ and metal-oxygen on the surface decreased, while the crystallite size increased. These factors led to a significant decrease in the activity of the catalysts. However, the sintering, crystal grain growth, and crystal phase change made its structure gradually compact, thus improving its mechanical strength. Although both Fe_2_O_3_-350 and RM350 contained a similar amount of surface Fe^3+^ and metal-oxygen, RM350 had a higher specific surface area and oxygen mobility, stronger component interaction, and a lower reduction temperature. These factors made the catalytic activity of RM350 much higher than that of Fe_2_O_3_-350. As shown in Table 4, compared with other biodiesel wastewater treatment processes, a higher COD removal rate was achieved using the catalytic combustion process without the addition of microorganisms and other chemical reagents such as strong oxidants.

## 4. Conclusions

In summary, a series of RM catalysts at different calcination temperatures were prepared by a simple method. Among them, the catalyst exhibited excellent catalytic activity as the calcination temperature did not exceed 400 °C. RM350 showed the best catalytic activity for the catalytic combustion treatment of biodiesel wastewater. The COD of the treated wastewater was 0 mg/L, and the COD removal rate reached 100%. The characterization results showed that calcination could increase the specific surface area of the RM catalysts to provide more active sites. As the calcination temperature did not exceed 400 °C, the catalyst had a highly developed pore structure conducive to adsorption and diffusion. Most of the Fe_2_O_3_ in the catalysts existed in an amorphous form, and the dispersion of α-Fe_2_O_3_ in the crystalline state was higher than that of other calcination temperature catalysts. The surface of the catalysts contained a large amount of Fe^3+^ and metal-oxygen, which significantly reduced the reduction temperature of the catalyst and enhanced its reducibility. As the calcination temperature exceeded 400 °C, the sintering of the catalyst and the increase in α-Fe_2_O_3_ crystallite size led to a rapid decrease in the specific surface area but increased the mechanical strength. The increase in α-Fe_2_O_3_ crystallite size also reduced the dispersion of Fe_2_O_3_. The results can provide some reference and ideas for the application of RM, the preparation of RM catalysts, and the catalyst selection in catalytic combustion.

## Figures and Tables

**Table 1:** Physicochemical characteristics of biodiesel wastewater.

Parameter	Unit	Value
COD (Chemical Oxygen Demand)	mg/L	114,500 ± 7600
BOD_5_ (5-day Biochemical Oxygen Demand)	mg/L	55,750 ± 3700
SS (Suspended Solids)	mg/L	59.3 ± 2
EC (Electrical Conductivity)	μS/cm	190 ± 5
Colority	times	11
pH	—	3.85 ± 0.05
Appearance	—	Yellow

**Table 2:** XRF analysis result of the RM element composition (wt%).

Element	RM	Error
Fe	27.74	0.17
Al	12.64	0.11
Si	10.42	0.09
Na	7.36	0.13
Ti	2.73	0.06
Ca	0.398	0.02
Mg	0.174	0.009
S	0.157	0.008
K	0.116	0.006
V	0.0998	0.005
P	0.0939	0.0047

**Table 3:** BET surface areas, pore volumes, average pore sizes, crystallite sizes, and XPS results of the as-prepared catalysts.

Sample	S_BET_ (m^2^/g) ^a^	V_p_ (cm^3^/g) ^b^	D_p_ (nm) ^c^	Crystallite Size (nm) ^d^	Fe 2p (eV) ^e^		O 1s (eV) ^e^	
					Fe 2p_3/2_	Fe 2p_1/2_	O_left_	O_right_
RM120	43.37	0.124	11.39	15	710.8	724.3	529.6	531.4
RM300	61.55	0.152	9.87	15	710.5	724.0	529.7	531.6
RM350	64.15	0.147	9.19	15	710.5	724.0	529.7	531.6
RM400	60.03	0.150	9.98	15	710.5	724.0	529.8	531.5
RM500	46.10	0.133	11.53	15	710.4	723.9	529.8	531.5
RM600	33.63	0.157	18.65	16	710.4	723.9	529.7	531.1
RM700	23.65	0.132	22.26	19	710.3	723.8	529.6	530.9
RM800	12.21	0.084	27.64	32	710.4	723.9	529.5	530.9
RM900	6.74	0.055	32.57	51	710.3	723.8	529.4	530.8
RM1000	0.80	0.005	22.47	>100	710.4	723.9	529.6	531.2
RM1100	0.65	0.005	32.83	88	710.3	723.8	529.5	531.0
Fe_2_O_3_-350	7.62	0.040	17.27	—	710.3	723.8	529.8	532.0

^a^ The BET surface area was determined by the linear part of the BET equation (P/P_0_ = 0.04–0.32). ^b^ The total adsorption pore volume at P/P_0_ = 0.99. ^c^ Average pore diameter. ^d^ The data were determined based on XRD results according to the Scherrer equation of fwhm using the (104) planes of α-Fe_2_O_3_. ^e^ The data were determined by fitting the XPS results with the Voigt function.

**Table 4:** Comparison of different processes for biodiesel wastewater treatments.

Process	Biodiesel Wastewater			References
	Original COD (mg/L)	Treated COD (mg/L)	COD Removal Rate (%)	
Catalytic combustion	109,159	0	100	This work
Membrane treatment	47,200	13,688	71	[10]
Electrocoagulation	399,800	35,982	91	[58]
Coagulation-flocculation and photolysis	5960	1311	78	[59]
Acidification and coagulation	60,000–150,000	10,000–20,000	80–90	[60]
Biological treatment	40,975	9793	76.1	[61]
Chemical coagulation	271,000–341,712	6480	97.6–98.1	[8]

## Data Availability

The original contributions presented in this study are included in the article. Further inquiries can be directed to the corresponding author.
